# Research Participant Bill of Rights: Clarifying the Role of Research Physicians

**DOI:** 10.1002/eahr.60035

**Published:** 2025-09-27

**Authors:** Mark A. Rothstein

**Affiliations:** ^1^ Director of translational bioethics at the Institute for Clinical and Translational Science at the University of California, Irvine

**Keywords:** human research ethics, clinical research, medical ethics codes, transparency, accountability

## Abstract

Physicians engaged in clinical research have dual roles as members of the research team and as physicians. Although various medical ethics codes and associated ethics opinions state that physicians have substantially similar obligations to research participants as they have to patients, the reality is different. Research physicians follow study protocols that often require practices that depart from the standard of care. The proposed Bill of Rights for Clinical Research Participants incorporates key ethics principles from the physician‐patient relationship, some of which already are reflected in provisions of the ethics codes and opinions addressing nontraditional physician relationships. The Bill of Rights is intended to promote greater transparency and accountability in a frequently misunderstood relationship.

Physicians play an indispensable role in clinical research, but one that often contains conflicting obligations and ethical responsibilities. As key members of the clinical research team, physicians advance the primary mission of research to discover generalizable knowledge. As medical professionals, they are also duty bound to “first do no harm” and to promote the well‐being of individuals with whom they have a professional relationship, even if it is not a traditional physician‐patient relationship.

Physicians conducting clinical research are mostly academic physicians and private practice physicians retained by pharmaceutical companies or research institutions. According to one study of private practice physicians in research, “physicians primarily envisage ethics in terms of adhering to the study protocols that pharmaceutical companies hire them to conduct.”^1^ It is also likely that many academic physicians performing research at large research institutions also view compliance with an institutional review board (IRB)‐approved study protocol as their primary ethical responsibility.

Physicians’ roles in clinical research vary widely, ranging from serving as principal investigators to merely examining potential participants to determine whether they meet inclusion criteria. Poorly understood or ill‐defined aspects of the relationship between research physicians and research participants can undermine both clinical research and the welfare of the participants. By clarifying and modestly supplementing the relationship through disclosures and minimum standards, research physicians can protect research participants from undue harm without undermining physicians’ ability to perform their essential role in clinical research.

Although it is arguable that all researchers ought to follow the ethical principles discussed in this article, physicians are the leaders of the medical component of clinical research and they are subject to detailed ethical precepts. The proposed Bill of Rights for research participants enrolled in clinical studies seeks to promote the interests of clinical research participants by harmonizing physicians’ traditional ethical obligations with the distinct demands of clinical research. It also seeks to supplement provisions in the Common Rule applicable to all researchers with more focused rights for participants when interacting with research physicians.

## RELATIONSHIP OF PHYSICIANS AND RESEARCH PARTICIPANTS

The possibility of discovering generalizable knowledge to improve human health is the ethical justification for research physicians to engage in practices that would often fail to meet the standard of care or conflict with ethical precepts of medical practice. For example, performing a biopsy, lumbar puncture, and imaging procedure is justified in clinical care only if the procedure contributes to diagnosis or treatment; but such procedures may be justified in clinical research when necessary to answer important scientific questions despite not being medically indicated.”^2^

Neither the Department of Health and Human Services research regulations (“Common Rule”)^3^ nor the U.S. Food & Drug Administration (FDA) research regulations^4^ address the physician‐participant relationship. IRBs and research funders routinely approve protocols with medical provisions that depart from the standard of care and principles of medical ethics applicable in practice settings. Many research protocols explicitly or implicitly apply different ethical standards to clinical research.

Notwithstanding the reality that different ethical standards apply to physicians engaged in clinical research, codes of medical ethics provide to the contrary—that the ethical obligations of physicians in clinical research are substantially the same as in clinical settings. Ethics Opinion 7.1.1 of the American Medical Association (AMA) states that physicians who are involved in research should “[d]emonstrate the same care and concern for the well‐being of research participants that they would for patients to whom they provide clinical care in a therapeutic relationship.”^5^

Similarly, paragraph 4 of the World Medical Association Declaration of Helsinki provides: “It is the duty of the physician to promote and safeguard the health, well‐being, and rights of patients, including those who are involved in medical research. The physician's knowledge and conscience are dedicated to the fulfillment of this duty.”^6^

The consequences of a divergence between customary research practices and codes of medical ethics are not merely semantic or academic. If participants regard research as a form of medical care, often referred to as “therapeutic misconception,”^7^ they might erroneously think that medical examinations or procedures during research eliminate the need for regular, comprehensive medical care. In addition, some patients in clinical trials who consent to undergo tests or procedures involving greater than minimal risk or discomfort may not realize that there may be little or no prospect of diagnostic or therapeutic benefit to them. Even more troubling, if physical examinations, laboratory tests, or imaging performed in research indicate a potentially serious medical problem, physicians might be prevented (or at least not encouraged or required) by some protocols from informing the research participant, thereby delaying essential medical care.

There has been little empirical research on disclosure of incidental findings in research. The most detailed study found that relatively few research guidance documents available on the internet from four main groups even mention incidental findings (federal government 9%; professional societies 25%; universities 11%; and web‐based consent forms 37%).^8^ Still fewer documents discuss whether to disclose incidental findings; of the ones that mention disclosure, more of them recommend disclosure than nondisclosure. The study did not review industry‐sponsored research and many research sponsors might perceive any medical disclosures to research participants as possibly revealing proprietary information.

Defenders of the current role of physicians in medical research can point to informed consent documents that describe the procedures and limited protections of research participation. Arguably, research participants freely accept all the conditions of participation in clinical trials. Numerous studies, however, have demonstrated that many participants do not carefully read, comprehend, or remember the contents of informed consent documents.^9^ The 2018 revisions of the Common Rule attempted to bolster informed consent by adding the requirement that researchers include “key information” in an understandable form and applying a “reasonable person” standard to determine what must be disclosed,^10^ but it is unclear whether these measures have been successful^11^ and whether they have achieved the goal of “rescuing” informed consent.^12^

In addition, many patients who participate in clinical research are significantly impaired by their illness, and even though they may be competent to consent, they might not completely appreciate the conflicting goals of research versus treatment. The substantial empirical research showing a deficit in knowledge transfer in the informed consent process clearly suggests that some or many research participants misunderstand important aspects of studies, including the atypical relationship between research physicians and research participants. Therefore, informed consent should not be considered a waiver by participants of all their rights and interests. Paragraph 9 of the Declaration of Helsinki states: “The responsibility for the protection of research participants must always rest with physicians or other researchers and never with the research participants, even though they have given consent.”^13^

## COMPARABLE RELATIONSHIPS

Although the relationship between research physicians and research participants differs from the traditional physician‐patient relationship, it is not unique. The terms “dual obligation,” “third‐party,” or “limited” physician‐patient relationship describe arrangements in which medical assessment or care is provided by or for the benefit of a party other than the individual whose health is at issue. Comparable relationships include medical evaluations of applicants or employees by employers,^14^ workers’ compensation examinations,^15^ military medical examinations and care,^16^ and medical services provided by physicians employed by professional sports teams.^17^

Of particular relevance and significance is the AMA's Ethics Opinion 1.2.6,^18^ which provides that physicians employed by businesses or insurance companies who provide medical examinations should (1) disclose the nature of the physician's relationship with the third party; (2) explain the difference between “this practice and the traditional fiduciary role of a physician”; (3) protect the individual's health information according to professional standards; and (4) inform the individual about incidental findings and, when appropriate, suggest follow‐up care and provide reasonable assistance in doing so.

These essential provisions of the AMA's Ethics Opinion 1.2.6 do not apply to the relationship between research physicians and research participants. Nevertheless, the disclosures and protections in this opinion may be even more important in research settings because individuals can experience a variety of physical and psychological harms in clinical research. By contrast, any harms from a medical examination conducted for an employer or insurance company are mostly economic, such as loss of employment or denial of insurance coverage.

## BILL OF RIGHTS FOR CLINICAL RESEARCH PARTICIPANTS

One way of aligning customary research practices with the ethical obligations of physicians expressed in the codes and opinions of medical ethics

**The Bill of Rights for Clinical Research Participants incorporates some of the important ethics elements of the physician‐patient relationship into research settings through a series of disclosures and minimum standards of conduct.**

would be to revise the codes and opinions to be more realistic and expressly state that common research practices are designed to generate new knowledge and not to protect the interests of participants.^19^ In theory, this approach might make informed consent more “informed,” but it would not necessarily change the way clinical research is conducted or improve the well‐being of participants. It would also dilute the aspirational goals of nonbinding codes and opinions regarding the beneficent role of physicians. Another option, explored below, is to incorporate a limited number of basic principles from the physician‐patient relationship into the physician‐research participant relationship.

Integration of key principles of medical ethics into clinical research can be achieved through essential disclosures and minimum standards. The Bill of Rights for Clinical Research Participants builds on the analogous responsibilities of physicians performing medical examinations for third parties described in the AMA's Ethics Opinion 1.2.6. Framing the research obligations of physicians as a Bill of Rights for Clinical Research Participants emphasizes the foundational nature of the principles and is intended to increase the awareness and understanding of them by research participants.

Other bills of rights applicable to research participants have been adopted or proposed. For example, in 1978, California enacted the Experimental Research Subject's Bill of Rights.^20^ This law, however, merely tracks rights contained in the Common Rule, such as the right to be informed about the nature of the research; the right to be informed about the risks, benefits, and alternatives to the research; and the right to informed consent.

By contrast, the Bill of Rights for Clinical Research Participants goes beyond the Common Rule and focuses on the physician‐participant relationship. Three of the provisions (3, 4, and 5) are intended to supplement the informed consent process mandated by the Common Rule^21^ by reiterating required elements of informed consent at a time and in a context where they are more likely to be comprehended by participants. The other provisions extend to the clinical research setting individual rights recognized in other contexts.

The 10 principles that encompass the proposed Bill of Rights for Clinical Research Participants (see table 1) are described below with accompanying explanations.

**Table 1:** Bill of Rights for Clinical Research Participants

***Clinical research participants have the following rights:***
To be told about the physician's employer, credentials, and role in the research. To be informed about how a physician's role differs in clinical practice and research. To be told about the nature and purpose of the medical procedures in the research. To be told about privacy, confidentiality, data security, and data sharing. To be asked to consent for each medical test or procedure and told about the right to decline or terminate any test or procedure. To be offered information about important incidental findings. To be offered to have medical information sent to the participant's personal physician. To be offered referral for medical follow‐up. To be offered to be recontacted if new medical information is discovered. To receive treatment and compensation for harms resulting from the research.

### Clinical research participants have the right to be told about the physician's employment, credentials, role in the research, and any conflicts of interest

At the initial encounter with each participant, physicians should disclose (1) their relevant employment relationships, such as employee of the research sponsor, member of the medical staff of a health care institution, or independent consultant; (2) their credentials, including degrees, licensure, specialty certification or training, and relevant experience; (3) their role in the research, such as principal investigator, co‐investigator, or consultant; (4) whether they will have a one‐time or continuing relationship with the participant during the study; and (5) whether they have any economic or other actual or perceived conflict of interest.

### Clinical research participants should be informed about how a physician's role differs in clinical practice and research

Physicians should inform participants that the traditional elements of the physician‐patient relationship do not fully apply in research designed to discover generalizable knowledge and should describe key aspects of the physician‐participant relationship. This involves contrasting the physician‐participant relationship in research with the more widely understood physician‐patient relationship in clinical practice. Participants also should be informed about the roles of nurses, study coordinators, technicians, and other members of the research medical team. The disclosures are intended to increase participants’ understanding and build trust.

### Clinical research participants should be told about the nature and purpose of the medical procedures in the research

In supplementing the Common Rule and FDA requirements for the informed consent process, research physicians should ensure that a member of the medical research team informs participants of the nature and purpose of medical procedures in the research, such as to measure the effects of investigational drugs on certain organs or systems.

### Clinical research participants should be told about privacy, confidentiality, data security, and data sharing

In supplementing the Common Rule and FDA requirements for the informed consent process, research physicians should ensure that a member of the medical research team informs participants of how individual health information will be used and disclosed in the study and whether anonymization, coding, encryption, or other methods will be used to protect confidentiality. Participants also should be informed about data retention policies and how health information may be shared with other researchers or entities for secondary research.

### Clinical research participants should be asked to consent for each medical test or procedure and be told about the right to decline or terminate any test or procedure

In supplementing the Common Rule and FDA requirements for the informed consent process, research physicians should ensure that a member of the medical research team affords participants the opportunity to consent for each medical test or procedure. Participants should be reminded that they have a right to refuse any test or procedure, but that if they refuse, they could be withdrawn from the study.

### Clinical research participants should be offered information about important incidental findings

Physicians should ask participants whether they want to receive information about important incidental findings discovered in initial or ongoing medical assessments in the research and their possible significance.^22^ This includes medically noteworthy incidental findings from physical examinations (e.g., extremely high levels of hypertension), laboratory findings (e.g., blood sedimentation levels well beyond reference range), and imaging (e.g., neoplasm noted on a CT scan). Minor anomalies need not be reported (e.g., borderline hypercholesterolemia), and some verification and consultation with medical colleagues may be required. In some extreme situations, emergency or at least expedited follow‐up medical care should be arranged. In their 2008 analysis of incidental findings, Susan Wolf and colleagues noted that “Research currently proceeds with no consensus that researchers have duties to analyze anomalies spotted, secure a clinical consult to verify the existence of IFs [incidental findings], and offer to disclose verified IFs of likely importance to the research participant.”^23^

### Clinical research participants should be offered to have medical information sent to their personal physician

Physicians should offer to send clinically relevant medical information to the participants’ personal physician or provide the participant with actionable, personal medical information from the study.^24^

### Clinical research participants should be offered referral for medical follow‐up

Where medically indicated, physicians should offer to inform participants of the need for medical follow‐up and offer to provide them with referral information and assistance. In some extreme situations, expedited or immediate care needs to be arranged. Referral information and assistance, including to a specialist, is especially important where the participant has not indicated a personal physician who can maintain continuity of care.

### Clinical research participants should be offered to be recontacted if new medical information is later discovered

Although research findings are generally not revealed until after completion of the study, such as via publication, individual results might have important and more immediate health implications. Participants should be able to elect notification about important information learned after completion of their involvement in the study and of relevance to their medical condition, such as the risks of a participant continuing to take their current medication. The use of electronic communication lessens the burden of contacting study participants in such situations.^25^ All clinical trial participants also have an interest in the general results of the study.^26^

### Clinical research participants should receive treatment and compensation for harms resulting from the research

The Common Rule^27^ and FDA^28^ requirements for informed consent provide that there must be an indication “whether” medical care or compensation will be provided in the event that a participant is harmed by the research, but there is no requirement for care or compensation to be provided. Despite repeated calls for compensation from national bioethics commissions and the Institute of Medicine,^29^ more than half of U.S. research institutions do not offer compensation and more than 20% of institutions include potentially exculpatory language in consent forms. The percentages changed little from 2000 to 2012, thus there is little evidence to suggest there is much difference today. By contrast, other countries overwhelmingly mandate care or compensation for individuals harmed in research, sometimes by researchers or research sponsors purchasing of insurance.^30^ Paragraph 15 of the Declaration of Helsinki plainly states: “Appropriate compensation and treatment for participants who are harmed as a result of participating in research must be ensured.”^31^ Although research sponsors and institutions rather than physicians are the appropriate parties to provide care and compensation, attaching an ethical obligation on physicians to disclose these measures could encourage their adoption.

## CONCERNS OF RESEARCH SPONSORS AND OTHER STAKEHOLDERS

The proposed Bill of Rights for Clinical Research Participants incorporates modest measures consistent with existing ethical principles. Nevertheless, it would change some current practices and research sponsors, researchers, and other stakeholders could have one or more of the following five main concerns.

First is the possible disclosure of proprietary information. However, only principle 9 of the Bill of Rights (disclosure of medical information) could involve information generated by the study and disclosures need not include proprietary information.

Second is that certain individual disclosures of health information (principles 6, 7, 8, and 9) will undermine the scientific integrity of the study by unblinding the identity of individual participants. Nevertheless, it is possible to maintain blinding of the study by separating medical team member responsibilities, such as assigning disclosure obligations to investigators who are not responsible for analyzing data.

Third is that physician disclosures would be time consuming. Principles 3, 4, 5, and 9 do not require direct physician involvement, and other personnel including research coordinators and nurses can be assigned to make these disclosures. The physician's involvement in satisfying the other principles should not take an inordinate amount of time. Furthermore, similar disclosures by physicians are currently required by medical ethics provisions applicable to medical assessments by third parties, such as examinations for life insurance coverage. Participants in clinical research are not merely examined but often have a degree of risk and discomfort and therefore deserve an appropriate level of medical disclosures. Of related concern, the modest changes associated with the Bill of Rights are unlikely to be financially burdensome on research sponsors, especially considering the substantial costs of clinical research.

Fourth is that if researchers assume a duty, for instance to disclose incidental findings, the failure to satisfy the duty could lead to legal liability. Yet, it is possible that there already could be liability for nondisclosure of incidental findings even without a physician‐patient relationship and despite inclusion of a disclaimer in an informed consent document.^32^

Fifth is the concern that compensation for research‐related injuries should not be available for patients whose existing medical condition worsened regardless of the research. However, as with any claim for medical injury, the claimant would need to prove a causal relationship between the clinical trial and the asserted injury, such as where the harm was of a different body part or organ system, or where similar adverse events occurred in other research participants.

## CONCLUSION

Many of the ethical problems associated with physicians engaging in clinical research involve a lack of transparency, fidelity, and beneficence—hallmarks of the physician‐patient relationship. Notwithstanding the absence of a traditional physician‐patient relationship and recognizing the important differences between medical care and research, physicians still have ethical obligations to research participants with whom they interact in a professional capacity. The essential obligation is to conduct research in a reasonable manner consistent with promoting transparency and protecting the well‐being of research participants.

The Bill of Rights for Clinical Research Participants incorporates some of the important ethics elements of the physician‐patient relationship into research settings through a series of disclosures and minimum standards of conduct. Several of these principles have been adopted by the AMA's ethics opinions addressing other nontraditional relationships, including employer‐retained physicians and independent medical examiners. The principles of the Bill of Rights for Clinical Research Participants can be adopted with a minimum amount of effort and while maintaining the scientific integrity of the study. Table 2 lists the various research ethics stakeholders who could adopt the Bill of Rights or, at the very least, endorse its key principles.

**Table 2:** Adoption of the Bill of Rights for Clinical Research Participants

***The Bill of Rights or its key principles could be adopted by:***
individual or groups of physicians commercial sponsors of research nonprofit sponsors of research government research funders health care and academic medical institutions research ethics regulators institutional review boards drafters of codes of medical ethics medical licensing boards state or federal legislators

The limited modifications of clinical research medical practices embodied in the Bill of Rights for Clinical Research Participants would not alter or undermine the fundamental goals, methods, or ethics elements of clinical research. Moreover, by establishing a robust, trusting, and meaningful relationship between research physicians and research participants, the Bill of Rights for Clinical Research Participants has the potential to advance scientific discovery by increasing the willingness of physicians and potential research participants to take part in important studies.

Some research sponsors and medical institutions already have adopted these principles through internally developed rules or IRB policies. The Bill of Rights for Clinical Research Participants is intended for use in the U.S., but other jurisdictions might find it a valuable starting point for their own versions.

## ACKNOWLEDGMENTS

Kyle Brothers, Simon Lucas, Gil Siegal, and Leslie Wolf provided valuable comments on an earlier draft.
